# Supplementary figures and images for: Clinical characteristics and serotype association of dengue and dengue like illness in Pakistan
Source: PLoS Negl Trop Dis. 2025 May 12;19(5):e0012978. doi: 10.1371/journal.pntd.0012978 (PMC12068610; doi:10.1371/journal.pntd.0012978)

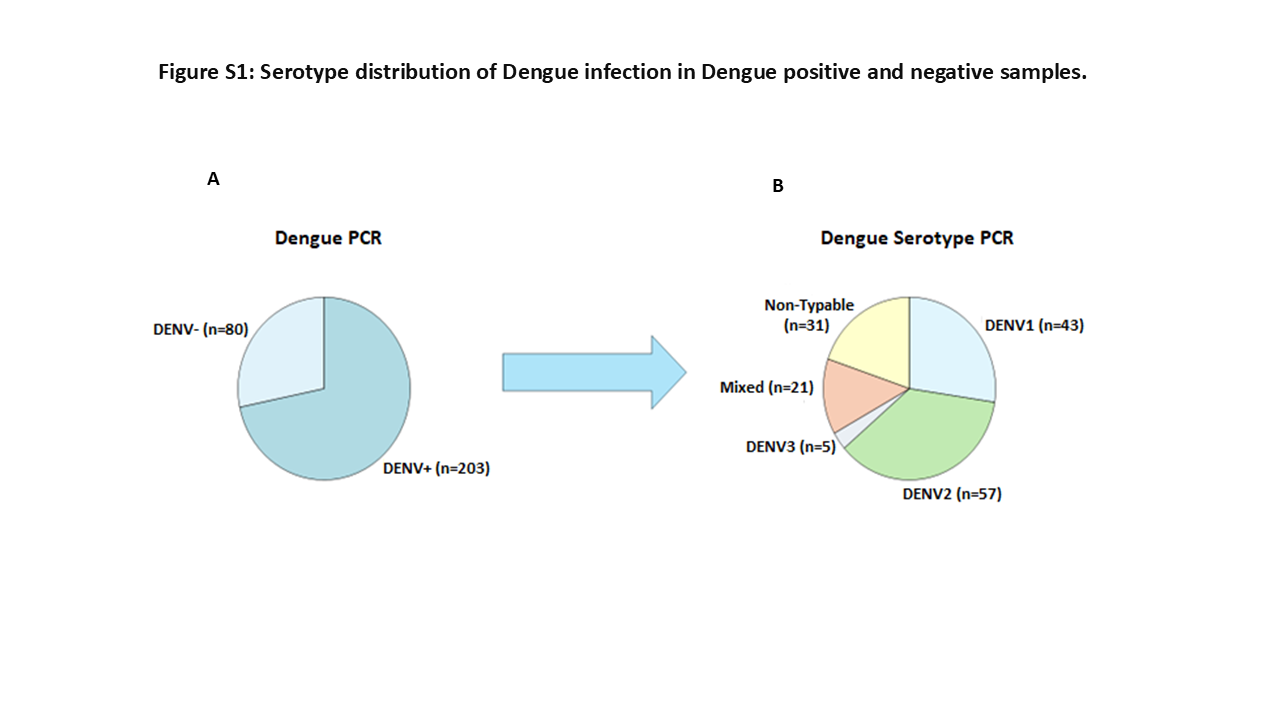

Supplement: S1 Fig — Dengue samples were subjected to PCR and serotyping using ZCD assay. (A) Out of a total of 283 samples, 203 (72%) were diagnosed as DENV+ and 80 (28%) as DENV− (B) Dengue serotyping of 157 DENV+ samples revealed a predominant classification into DENV 1 (n = 43), DENV 2 (n = 57), and DENV 3 (n = 5) with mixed serotypes (n = 21) [DENV 1 | DENV 2 (n = 20) and DENV 1 | DENV 3 (n = 1)] detected in 13% of cases. (TIF) [file pntd.0012978.s001.tif]

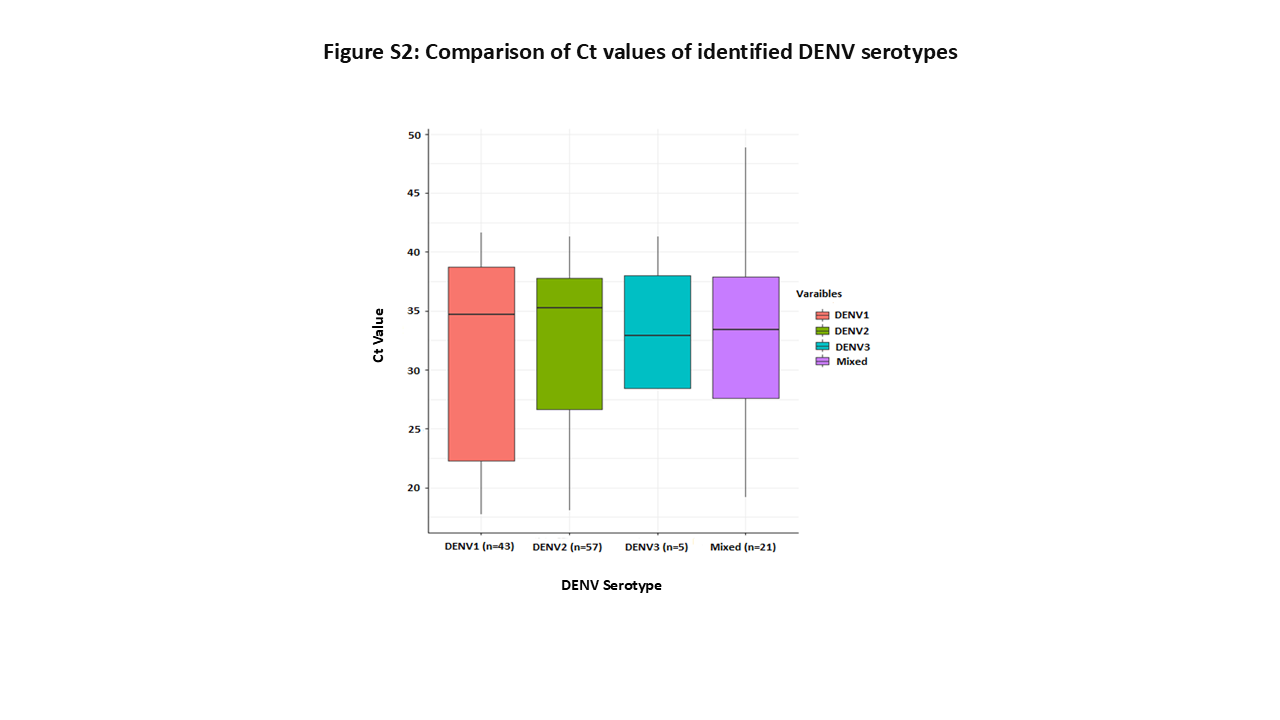

Supplement: S2 Fig — q-RTPCR Ct Value distribution in dengue serotypes. No significant differences in Ct values were observed between DENV1, DENV2, DENV3 and mixed serotypes. (TIF) [file pntd.0012978.s002.tif]

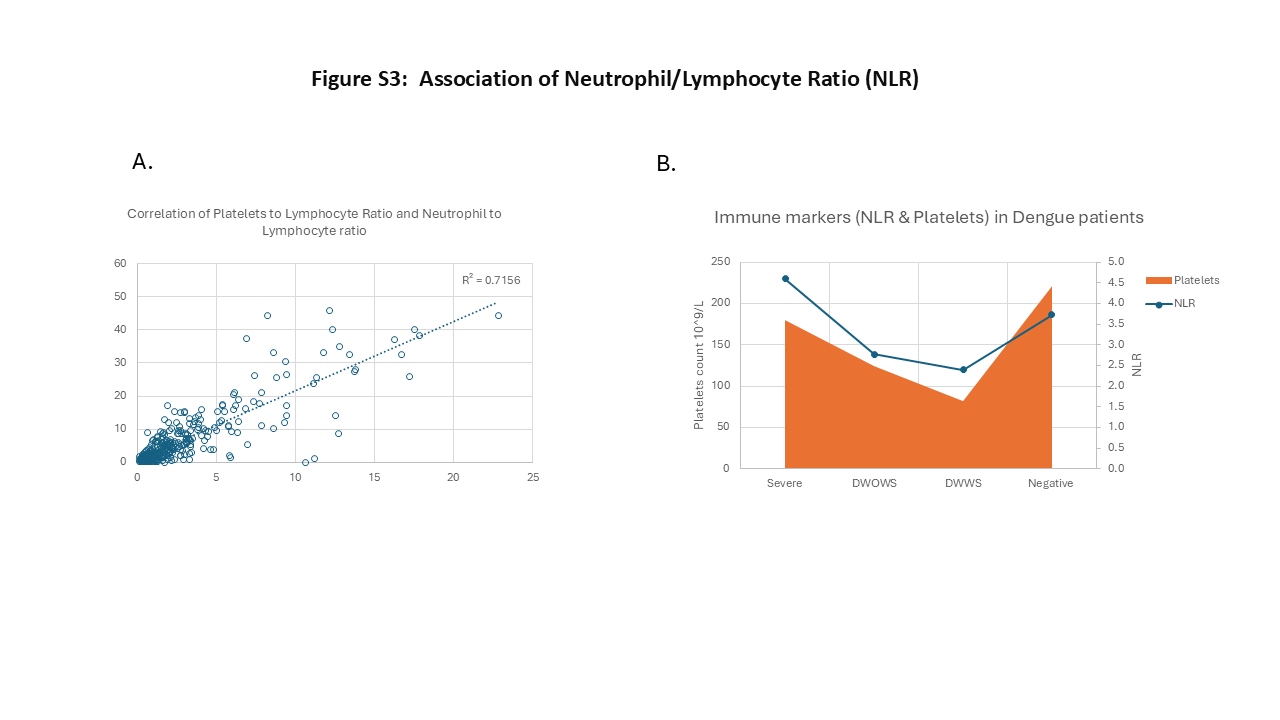

Supplement: S3 Fig — NLR and PLR show a high positive correlation in patients with Dengue, (B) NLR and platelets count in pateints with Dengue severity. (TIF) [file pntd.0012978.s003.tif]

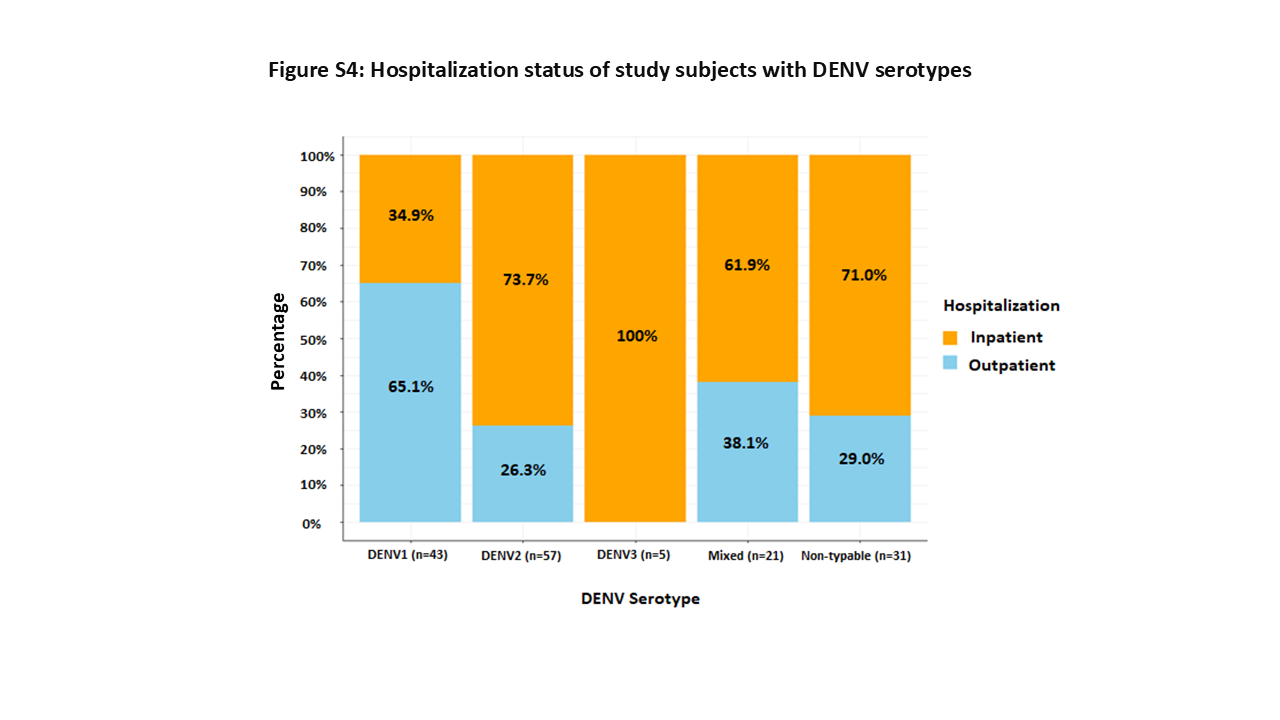

Supplement: S4 Fig — Hospitalization status of Serotype-specific participants. High percentages of DENV2, mixed serotypes and non-subtype participants were in-patient. (TIF) [file pntd.0012978.s004.tif]
