# Supplementary material for: Clinical characteristics and serotype association of dengue and dengue like illness in Pakistan
Source: PLoS Negl Trop Dis. 2025 May 12;19(5):e0012978. doi: 10.1371/journal.pntd.0012978 (PMC12068610; doi:10.1371/journal.pntd.0012978)
Supplement: Attachment — Submitted filename: PLoSNTD-25Feb2025-Response to the reviewers_Final.docx [file pntd.0012978.s008.docx]

Date: 26-02-2025

Dear Editor

Thanks for giving us this opportunity for re-submission of our revised article, Title: Clinical characteristics and serotype association of dengue and dengue like illness in Pakistan. We have revised results and discussion of the current manuscript and responded reviewer’s comments point wise in the table below.

Response to the reviewers

| Comments | Response | Line#, Page # |
| --- | --- | --- |
| Reviewer #1: the objectives of the study clearly articulated with a clear testable hypothesis, and the study design appropriate to address the stated objectives. the sample size sufficient to ensure adequate power to address the hypothesis  Reviewer #2: A minimum sample size should be defined using statistical methods, this was ommitted.  Statistical methods to assess sensitivity and sensibility were not included, which was the gold standard? A detail statistical methodology must be included, and a valid scientific argument of using the selected gold standard according to the nature of the diagnosis of dengue. | Thanks for highlighting this point. It is a convenient sampling design and there was no testable hypothesis for this work. It was a hospital surveillance study designed to capture Dengue viral surveillance in a hospital and out-patient clinics. *The sample size was determined by availability of data during the data collection process which was started in June 2021 and continued during the year 2023, but data was included in this manuscript was collected until November 2023. Detail of 349 samples are mentioned in Supplementary Appendix I.* The exact prevalence of Dengue is not known in Pakistan. On average 25,932 cases were reported between Jan -Sep 2022 (<https://www.who.int/emergencies/disease-outbreak-news/item/2022-DON414>) ^1^  For a hospital surveillance study in a single city of Karachi, a sample size of 349 was adequate to capture true representation of cases reported in Sindh between 2021 to 2023.  **Gold Standard:** All NS1+ and IgM+ OR either of NS1+ / IgM+ were used as “Gold Standard”.  For ROC analyses, all “Final Diagnosis” positive including lab confirmed diagnosis and clinical diagnosis were included as Dengue positive Case (n=283).  This has been revised in the manuscript.  *“The sensitivity of q-RT PCR was calculated based on the clinical diagnosis. The sensitivity of q-RT PCR was calculated based on the case definition used for Dx positive (Dx+) cases which comprised of both NS1+/IgM+, NS1 OR IgM positive OR clinical presentation of “Dengue fever”, “Dengue suspects”, “Dengue like illness” and all “viral fever”. This constitutes a total of 349 cases which include Dx+ (n=262) [NS1+/IgM+/Clinical Dx+] and Dx – (n=87) [NS1-/IgM-/Clinical Dx-] groups”*  *In addition to the above analyses, sensitivity and specificity were also calculated for the Gold standard positive samples (NS1+ OR IgM+ OR NS1+ and IgM+) which include 267 cases excluding strong clinical suspicion of Dengue fever (n=16). The qRT-PCR sensitivity (86.63%; CI:80.90% to 91.16%) and specificity (67.50%; CI: 56.11% to 77.55%) remained same with minor differences in AUC of 0.773 [95% CI: 0.705 to 0.840] Table S4 b&c*. | 106-109 page #5  202-210  Page# 9 |
| Reviewer #1: the results are clearly and completely presented  Reviewer #2: The title 3.2 sensitivity of serological pattern has no relationship with the presented results, as in the results described the sensitivity of clinical diagnosis compared to PCR.  3.5 Clinical Diagnosis of Dengue Mixed Serotypes: Is it possible to present this results in a table, for a better understanding of the data  Figures are not of sufficient quality | Thanks for identifying this typo: it has been corrected to *“Sensitivity of molecular diagnostics for dengue Infection”*  Table 5 has been added in the manuscript which compares 21 patients including 13 in-patient and 8 out-patient samples and their clinical features  Resolution as per journal requirement | Page# 14  Table # 5 |
| Reviewer #1: the current study conducted in a large tertiary care centre in Pakistan, highlights the profound impact of Dengue worsened by flooding and poor sanitation, posing considerable challenges to public health. The high prevalence of Dengue cases emphasizes the urgent need to address the importance of robust infrastructure and sanitation. Immediate measures by authorities are crucial to control vector populations and protect public health. The main limitation of this study includes lower number of dengue severe cases and the unavailability of the resources to perform dengue sequencing for mixed infections.  Reviewer #2: Do the authors discuss how these data can be helpful to advance our understanding of the topic under study? The results and the data obtained, were no use correctly to discuss it -Is public health relevance addressed? It should be expanded and improved in the discussion | We agree with reviewer 1 about unavailability of sequencing results at this point of time in the current manuscript, however we are in the process of developing in-house sequencing capacity.  The study is relevant for our setting, where in an outbreak setting, a big question of serotype dominance in a particular outbreak was answered. Our data highlights a snapshot of 2021-2023 cases reported in a tertiary care setting. There was no change in the Dengue serotype prevalence compared to previous years^2^. The public health relevance for this data is the identification of a number of cases with Dengue fever presenting with atypical presentation of Dengue (n=41) (Suppl Appendix I, Sheet B). Samples with other diagnosis (NS1-/IgM-/IgM NA) cases being treated in our setting were picked by molecular diagnostic PCR, which gives a clear message to General health practitioners about low sensitivity of mainstream diagnostic tests such as NS1 and IgM tests. In cases with atypical presentation, clinical management of patients is important. Of note, these patients were hospitalized in the later stage with complications. | Line # 360-366  Page 16,17 |
| Reviewer #1: The current study aims to explore the clinical presentations and features of dengue fever in a tertiary care hospital. Collected data on cases including clinical symptoms and laboratory results including qRT-PCR and serotype characterization. A majority of subjects enrolled (75%) had mild disease without warning signs. The sensitivity of clinical diagnosis was found to be 87.25% and the specificity of 68.35%. qRT-PCR detected 43.5% of cases with viral fever initially screened negative. Screening with rapid tests requires further confirmation by molecular assay in cases with dengue and dengue-like illness.  Reviewer #2: A major revision is needed. The main concern is how the sensitivity and specificity results are presented, a rationale for the use of the gold standard is not detailed, and the methodology is not detailed. The discussion is brief, the data was used very little. The discussion should be expanded; nothing is discussed about the clinical findings and the serotypes found. | The sensitivity and specificity of qRT-PCR has been calculated by case definition (Table 4 and S4a ) and Gold standard (*Table S4 b&c*)  The sensitivity and specificity of qRT-PCR has been calculated by case definition (Table 4 and S4a ) and Gold standard (*Table S4 b&c*)  Discussion has been revised with relevance to clinical parameters and serotype differences. A statement on public health perspective has also been added  A new supplemental figure S3 has also been added on NLR  Table 5 and Fig S3 has been added in the revision | Line# 207-210 Page# 9  Line# 360-366, page #16-17 |
|  |  |  |

References:

1.	Khan U and Azeem S. The rising toll of dengue cases in Pakistan every year: An incipient crisis. *Ann Med Surg (Lond)*. 2022;76:103549.

2.	Umair M, Haider SA, Rehman Z, Jamal Z, Ali Q, Hakim R, Bibi S, Ikram A and Salman M. Genomic Characterization of Dengue Virus Outbreak in 2022 from Pakistan. *Vaccines*. 2023;11:163.
